# HIV-1 Virological Synapse: Live Imaging of Transmission

**DOI:** 10.3390/v2081666

**Published:** 2010-08-12

**Authors:** Jerome Feldmann, Olivier Schwartz

**Affiliations:** Institut Pasteur, Virus and Immunity Unit, URA CNRS 3015, 28 rue du Dr. Roux, 75724 Paris, France

**Keywords:** HIV-1, virological synapse, live video-microscopy

## Abstract

A relatively new aspect of HIV-1 biology is the ability of the virus to infect cells by direct cellular contacts across a specialized structure, the virological synapse. This process was recently described through live cell imaging. Together with the accumulated knowledge on cellular and molecular structures involved in cell-to-cell transmission of HIV-1, the visualization of the virological synapse in video-microscopy has brought exciting new hypotheses on its underlying mechanisms. This review will recapitulate current knowledge with a particular emphasis on the questions live microscopy has raised.

## 1. Introduction: HIV-1 cell-to-cell transfer

Most viruses infect new target cells, and new hosts, by means of cell-free viral particles. These particles can efficiently diffuse in the extra-cellular space and spread infection at a spatial and temporal distance. However, diffusion of viral particles lowers their concentration and increases the length of time between viral assembly and productive infection. Free virus spread thus requires both a sufficient half-life of the viral particle and sufficient infectivity. On the other hand, the immune system is built on a large network of cells in constant interactions with one another and migrating to almost every tissue. Some lymphotropic viruses like HIV-1 have taken advantage of this, developing means of transfer directly between cells. HIV-1 cell-to-cell transfer was noticed in the early 90s and it was immediately associated with a higher infectivity potential than cell-free virus *in vitro* [1–3]. Later studies confirmed the high efficiency of cell-to-cell transfer [4–7]. The observation that viral replication is impaired in shaken lymphocyte cultures strongly suggests that virus propagation is in large part dependent on cellular contacts [8]. Several observations argue for the importance of HIV-1 cell-to-cell transfer *in vivo*. In chronic infection, HIV-1 replication takes place mainly in lymphoid tissue, densely populated by T lymphocytes. 90% of infected cells at this stage are CD4+ T cells [9]. The mean number of integrated proviruses in splenic T cells is 3.2 [10], suggesting that most infected cells have received high virus concentrations. In addition, different viral quasi-species can be found in separated germinal centers of the same spleen, in different organs and in different cell types [11,12]. Viral replication therefore takes place mainly in cell-rich tissues where it seems to be locally constrained and highly efficient. This is more compatible with a cell-associated transmission rather than a systemic spread by free virus.

Several means of cell-associated transfer of HIV-1 have been discovered involving different cellular contexts. Uninfected cells can capture HIV-1 virions and transfer them to interacting lymphocytes [13–16]. The most spectacular case of this type is the trans-infection of T lymphocytes by uninfected dendritic cells (DCs) [14,17,18]. Monocyte derived DCs can capture HIV-1 virions and store them in plasma membrane invaginations without becoming infected [17,19,20]. Upon contact with a T cell, captured HIV-1 is relocalised to the site of formation of the immunological synapse (IS), and transferred to the T cell [21]. The hijacking of the IS by the virus to spread was termed the infectious synapse.

This review focuses on HIV-1 transfer from infected cells. Several modes of cell-to-cell transfer have been described and visualized by live imaging. The infected cells can contact and transfer virus to target through an extensive junction, the virological synapse [4,22,23], through filopodial bridges [24] or through nanotubes [25]. The precise contribution of these different modes of contact in viral spread *in vivo* is not known. *In vitro* however, the predominant form of contact is the virological synapse [7,23].

## 2. Structure of HIV virological synapse

Jolly *et al.* first described the HIV-1 virological synapse (VS) as the polarization of viral material between an infected Jurkat T cell and CD4+ primary targets [4]. Upon contact between the two cells, a rapid recruitment to the cell–cell contact surface of CD4, CCR5 or CXCR4, talin, actin and LFA-1 on the target cell is observed. Simultaneously, Env and Gag are recruited to the site of cell contact in the donor cell together with both lipid raft marker such as GM1, CD59, Thy1 and tetraspanins (CD63, CD81 and CD9) [23,26]. In T lymphocytes, HIV-1 budding takes place in cholesterol enriched lipid rafts [27] but also in tetraspanin enriched micro-domains [28]. Interestingly, the tetraspanins are modulated by HIV-1 and seem to act in preventing cell-cell fusion during cell-to-cell transfer [29,30]. Both tetraspanins and lipid rafts are polarized to the VS, indicating that budding zones are accumulating at the contact site. Accordingly, electron micrographs of the VS show both mature and budding virions at the contact site [22,23,31]. The VS may adopt a ring or button shaped structure, forming an interface between the infected and the target cell. Moreover, we observed both in primary cell and in Jurkat cells, that one infected cell may form synapses with up to five targets, in a structure we called a polysynapse [23].

## 3. Live transfer

**Figure 1.**
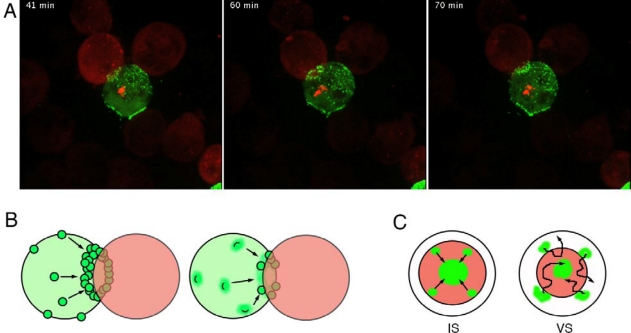
**(A)** The flexible structure of HIV-1 VS. Live imaging of Gag-GFP (green) infected cell expressing centrin-RFP (red dot), conjugated with a target expressing actin-RFP (red). Time from mixing of infected cells with targets is indicated. See complete movie at http://www.pasteur.fr/ip/portal/action/WebdriveActionEvent/oid/01s-00003u-006. **(B)** Models of VS formation. Mature virions can “surf” on the infected cell surface (left), or viral budding platforms can polarize toward the target (right). **(c)** Dynamic observation the IS and the VS reveals different behaviour of the two structures. This schematic view of the interface between interacting cells shows intercellular adhesion zones (pSMAC) in red, and mobile elements (TCR clusters in the IS, viral clusters in the VS) in green. TCR micro-clusters appear in the pSMAC of the IS then migrate centripetally to the cSMAC (left). Preformed surface viral material migrates uncoordinatedly toward the center of the VS (right).

Recently, VS formation as well as virus transfer were observed in live cells by time lapse video-microscopy [22,23], using GFP tagged viruses to visualize Gag movements. To minimize the perturbation of viral replication, the GFP was inserted between the matrix and capsid (CA), with cleavage sequences for the viral protease on both sides of the GFP or only between the GFP and CA [7,32]. These viruses, although impaired in their fitness, can be rescued by cotransfection of a GFP-free provirus. In addition, the localisation of Gag-GFP is similar to natural Gag in infected cells [7,32]. In infected HeLa [33] or T cells (Jurkat or primary) [23], Gag is visible as patches at the plasma membrane. Hubner *et al.* described the participation of these Gag patches to the formation of the synapse by lateral movements [22]. Interestingly, patches located around formed synapses tend to disappear, suggesting that membrane domains close to the synapse are preferentially recruited [22]. While Hubner and al. describe a button shaped synapse resembling the cSMAC [22], the synapses we observed were often circular and more evocative of the pSMAC of the IS [23]. A diversity of structure, including ring or button shapes, was similarly described for the IS and HTLV-1 VS [34,35]. Live imaging shows that the VS can alternate between ring or disk shapes (Figure 1), suggesting that the VS is relatively flexible. Proximal Gag patches behave thus as independent membrane domains that are attracted to the growing synapse. When they contact the synapse, they can either continue their lateral movements or are retained and merge with already accumulated patches. Although it is not always the case, the synapses can form relatively rapidly after contact, in approximately 15 minutes [7,23,31]. We showed that this Gag accumulation can occur at several contact sites simultaneously, and leads to efficient transfer to several target cells [23]. Together with the flexibility of the VS, this questions the necessity of the full polarization of infected cells for HIV transfer. Further work is required to determine whether one single infected donor cell can become multipolar, or whether the polarization is sequential toward each target cell. Viruses are known to subvert preexisting cellular processes at their advantage. “Immunological polysynapses” may thus also be operative. Indeed, CD4 T lymphocytes may form multiple IS with APCs, then polarize toward the highest antigen concentration [36]. In this context, the T cell response will probably be orientated by the polarization, but signal integration from multiple IS may play an important role in both the quality and the strength of the response. Additionally, cytotoxic T lymphocytes are able to mobilize lytic granules toward several targets simultaneously, eventually killing them [37–39]. Inter-cellular communication through multiple contacts therefore may represent an underestimated phenomenon [40].

The precise nature of the Gag patches in T cells, in the absence of intercellular contacts has not been fully explored. They might represent budded viruses bound to the cell surface, as observed by correlative electronic microscopy images of the zones of contact between infected cells and target cells [23]. These structures could also embed cellular proteins, or components of the extracellular matrix, as recently described for the biofilm-like structures induced by HTLV-1 [41]. The capture of large Gag-containing aggregates by the target cells, as observed by video-microscopy [22], suggests that virions may remain associated together after transfer. However, aggregated virions are not usually found in electron micrographs of the plasma membrane of infected cells. A notable exception is represented by the effect of tetherin. This interferon (IFN)-induced protein promotes the aggregation of mature virions at the cell membrane, impairing their release [42–44]. It is antagonized by the HIV-1 protein Vpu. In cells infected with Vpu-defective HIV-1, the virus is found in large aggregates at the cell surface. These large viral aggregates are transferred to targets during intercellular contacts, but then display reduced infectivity [45].

Similar Gag patches, corresponding to multimerized Gag proteins, are observed in fibroblastic cell lines [33,46]. Both membrane-bound and cytosolic Gag proteins are recruited to these patches. While Gag multimerizes relatively rapidly (virion formation occurs in 5–9 min), assembly sites form patches at the plasma membrane that can remain visible for 2 hours [47]. This is probably due to the limiting step of membrane fission, controlling viral egress [33]. A plausible model drawn from the live microscopy experiments is that Gag accumulates in budding platforms under the plasma membrane of infected cells [33,47]. Upon contact with a target cell and conjugate formation, proximal budding platforms are actively mobilized toward the contact zone, where they are stabilized by Env/CD4 interactions. Indeed, in live experiments, conjugates between infected and target cells can persist for at least two hours [22,31].

It is also noteworthy that live imaging revealed important differences between immunological and virological synapses. While the microclusters of TCR in the IS move unidirectionally toward the center of the synapse [48], Gag patches movements are more erratic and they can come out of the synapse (Figure 1c) [22]. This suggests that whereas the centripetal actomyosin flow is the main drive of the TCR microclusters, Gag patches are mobilized mainly through other mechanisms. A schematic diagram comparing components of the VS and the IS can be found in other reviews [49].

Live imaging was also used to describe HIV-1 transfer from infected monocyte derived macrophages (MDMs) to target cells [50]. In infected primary macrophages, HIV-1 virions can be found in large tetraspanin-enriched endosome-like structures [50–52] that are connected to the plasma membrane [51,53,54]. The follow-up of Gag-GFP showed that the protein is first targeted to the plasma membrane and then can be eventually internalized by invagination [55]. Upon contact with T cells or other macrophages, infected MDMs can efficiently relocalize the accumulated virus toward the forming synapse, allowing its transfer to the target [31,50,56]. The mechanism of VS formation, as revealed by live imaging, therefore seems very similar in macrophages and T cells: tetraspanin-enriched budding zones are relocalized rapidly at the site of contact with the target, although these budding zones are invaginated in macrophages. Intriguingly however, Gousset *et al.* showed that VS formation by infected MDMs can be Env independent, conversely with what is seen in infected T cells [4,50]. Polarization and transfer of viral material from infected MDMs to CD4 negative cells was independently observed [57]. This suggests that the signals provided by the intercellular contacts between MDMs and T cells may be stronger and sufficient to promote synapse formation, whereas between two T cells additional adhesion forces or signals, provided by the envelope may be required. The facilitating role of adhesion molecules such as LFA-1 in HIV-1 T cell to T cell transfer is established [23,58–61]. Their role could be even greater in MDM to T cell HIV-1 transfer. A study also documented viral spread from infected monocyte derived dendritic cells (MDCs) to T cells, which seems similar to macrophage transfer to T cells [62].

Live imaging also showed the transfer of HIV through filopodia and nanotubes. Nanotubes can be defined as extensions of the VS. They are Env dependant fine membrane cytonemes which connect the plasma membrane of infected and target T cells without necessarily connecting their cytoplasm [25]. As in the VS, Gag, Env and CD4 are accumulated in the contact zone between the projections and viral particle are transferred to the target from this site [25]. Furthermore, nanotubes can be detected after separation of infected and target cells [25]. Thus they might represent “stretched synapses” which allow prolonged contact even if the cells are migrating. Viral transfer through filopodia uses apparently a somewhat different mechanism. Plasma membrane projections, emitted by the target cell, attach to the infected cell through Env-CD4 interaction [24]. Viruses then surf along the filopodia toward the body of the target cell using an actin-based retrograde flow of receptors [24]. Spontaneous filopodia formation seems moderate in T cells [23,63]. However DCs and macrophages frequently form high numbers of filopodia [64,65] and this mean of viral spread could be an important process for HIV capture by these cells. Furthermore, HIV-1 infection reduces cellular motility [63], thus the ability of targets to contact infected cells at a distance through filopodia may be important for the spread of the virus *in vivo*.

## 4. Mechanisms of synapse formation: kinases, polarized budding and Env targeting

Relocalization of viral proteins, adhesion molecules and cellular receptors is relatively rapid, with 40% of the conjugates showing polarized synapses in 10 min [4]. This rapid recruitment suggests an active transport of receptors and viral proteins rather than a passive diffusion. The active mobilization of viral proteins is confirmed by the implication, in donor cells, of a signaling molecule, the ZAP-70 kinase, [66]. ZAP-70 is a central element in TCR signal transduction, required for actin remodeling during the formation of the immune synapse [67,68]. Our laboratory showed that a functional ZAP-70 is required in donor cells for polarization of viral material at the synapse and subsequent infection of targets. The exact role of kinase signals during viral cell-to-cell transfer is not fully understood. Whether ZAP-70 regulates the dynamics of the actin and microtubule skeleton [7,23,69], or exerts additional effects during synapse formation and virus transfer, will require further studies.

The first studies on the mechanism of cell associated transfer of HIV proposed a polarized secretion or budding of virions in MOLT T cells, stimulated by the interaction with plastic or epithelial cells [70]. Colchicine, a microtubule cytoskeleton inhibitor, induced the formation of an actin rich pseudopod-like structure where HIV-1 budding was concentrated [71]. Since then, polarized budding has been observed in monocytes [72] and in T cell lines at the uropod of motile cells [7]. A spontaneous polarization of viral material has also been described in infected Jurkat cells, which is dependent on intact actin and microtubule network [4,69,73]. The VS formation could then be seen as a relocation of this polarized budding zone at the site of contact with the target cell. As mentioned above, the IS induces the formation of membrane domains, which could favor the concentration of budding zones. The formation of these domains during VS transfer has not been explored yet.

Interestingly, the polarization of budding is associated with the sorting YXXL membrane proximal motif of gp41 [73]. This motif is responsible for Env cycling between the plasma membrane and the Trans-Golgi network [74] and is required for optimal infectivity [75,76]. In polarized epithelial cells, Env targeting directs HIV-1 budding to the baso-lateral membrane [77]. The Env/CD4 interaction is essential for the formation of the synapse [23]. Env could be targeted to the contact site, maintained there by its interaction with CD4 on the target, and attract Gag to form new virions. This mechanism has recently been described for cell-to-cell transfer of MLV in epithelial cells in which the cytoplasmic domain of Env directs viral assembly toward intercellular contact zones [78].

Tetraspanins are incorporated in HIV-1 virions [79] and enriched in budding areas [28,80,81]. They are linked to the actin cytoskeleton via EWI-mediated binding to ERM proteins [82]. ERM proteins (Ezrin-Radixin-Moesin) provide a dynamic link between plasma membrane proteins and the actin cytoskeleton [83]. Through their ability to interact with transmembrane proteins, phospholipids, membrane-associated cytoplasmic proteins and the cytoskeleton, ERMs organize complex membrane domains [84]. During IS formation, CD45 is excluded from the SMACs through Moesin mediated translocation [85] and Ezrin appears to mediate ZAP-70 recruitment to the synapse [86]. All together, this suggests an attractive potential model in which HIV-1 budding zones may be defined through tetraspanin and ERM proteins regulation.

Formation of VS is thus a complex process, regulated through numerous viral and cellular interactions.

## 5. Virological synapse and transfer efficiency

The assembly of a budding platform directly in contact with the target cell, the local production and transfer of fresh viruses, which are much more infectious than virions accumulating in the extracellular milieu, will enhance the efficiency of viral spread. Other mechanisms also enhance infectivity at the synapse. For instance, using cell-free viruses, it has been estimated than more than half of virions bound to target cells dissociate in 15 min [87]. The VS probably offers a stable framework, allowing the virus to remain in contact with the target cell for extended periods of time.

HIV-1 transmission across VSs has raised a great interest because of the potential implications on viral pathogenesis and drug resistance. In a particular setting, this process was found to be resistant to neutralizing antibodies and to a coreceptor antagonist [7]. However coreceptor independent capture of virions by uninfected targets does not necessarily reflect the subsequent infection of targets. Thus, while the first step of cell-to-cell transfer is resistant to inhibitors [88], the productive infection of the target is not [89]. An early study found that cell-to-cell transfer is more resistant to neutralizing antibodies and to AZT than infection with cell-free virus [90]. However, more recently, Martin *et al.* showed that the two modes of productive infection are similarly sensitive to neutralizing antibodies or entry inhibitors [31]. According to them, pre-formation of the synapses did not significantly increase the resistance to inhibition [31]. Electron tomography of the VS revealed a porous structure, allowing for diffusion of soluble molecules [31]. By contrast, the structure of the HTLV-1 VS shows extensive, close apposition of the membranes of infected and target cells [91]. It is also noteworthy that HIV cell-to-cell transfer may allow the virus to escape innate immunity. Indeed, type I IFN inhibits cell-to-cell transfer, but only partially, and less efficiently than infection by free viral particles [45,92]. Therefore, transfer across VSs seems to increase efficiency of infection, thereby inducing a partial resistance to antiretroviral molecules.

## 6. Conclusion and Perspectives

Live imaging allowed a direct real-time visualization of HIV-1 cell-to-cell transfer and demonstrated that the virus uses this very efficient mean to spread between cells. Multiple and complementary mechanisms of VS formation are probably operative. Assembly and budding may be promoted by intercellular contacts, but preassembled Gag patches are also “attracted” at the VS. Some cellular proteins involved in these processes have already been identified. Further work will be required to elucidate the full chain of molecular events controlling cell-to-cell transfer across the synapse. For example, ZAP-70 signaling is important for VS formation, but the other molecules involved, both upstream and downstream of ZAP-70, are still unknown. It will be also of particular interest to determine whether the constitution of polysynapses, involves specific mechanisms. Other modes of viral transfer through intercellular contacts include nanotube-like structures and filopodial bridges [24,25]. A dynamic visualization of the links that may exist between these various means of viral spread may also be obtained by live imaging. The study of HIV-1 synaptic transfer by video microscopy can be pushed further. In particular the site of viral fusion has not been convincingly demonstrated. Recent studies suggest that the virus does not fuse efficiently at the plasma membrane, but may have to be internalized first [93–95]. Using combinations of tagged proteins during synaptic transfer may reveal whether virions are internalized or can fuse directly at the plasma membrane. The effect of neutralizing antibodies and other inhibitory molecules on viral transfer and/or productive infection could also be visualized in real-time imaging, and may prove useful to understand their mode of action.

The next great challenge will be to analyze cell-to-cell viral transfer *in vivo*. In mucosal or lymphoid tissue of SIV infected monkeys, the virus infects clusters of cells, strongly suggesting that it is spreading through cellular contacts as described *in vitro* for HIV-1 [23]. However, virological transfer through synapses, filopodia or nanotubes remain to be observed *in vivo*. The IS is starting to be described at the molecular level, using two photon microscopy, in living mice [96]. The movement of viral particles, and the formation of VS, might also be soon visualized in animal models, using for instance humanized mice or monkey tissues [97–99]. Visualizing the spread of the virus during acute infection and AIDS progression in whole animals would be particularly interesting and could reveal the key mechanisms of the establishment of the infection and the immunodeficiency.
